# The charm-quark contribution to light-by-light scattering in the muon $(g-2)$ from lattice QCD

**DOI:** 10.1140/epjc/s10052-022-10589-2

**Published:** 2022-08-02

**Authors:** En-Hung Chao, Renwick J. Hudspith, Antoine Gérardin, Jeremy R. Green, Harvey B. Meyer

**Affiliations:** 1 PRISMA+ Cluster of Excellence, Institut für Kernphysik, Johannes Gutenberg-Universität Mainz, 55099 Mainz, Germany; 2 Aix Marseille Univ, Université de Toulon, CNRS, CPT, Marseille, France; 3 School of Mathematics and Hamilton Mathematics Institute, Trinity College, Dublin 2, Ireland; 4 Helmholtz Institut Mainz, Staudingerweg 18, 55128 Mainz, Germany; 5 GSI Helmholtzzentrum für Schwerionenforschung, Darmstadt, Germany

## Abstract

We compute the hadronic light-by-light scattering contribution to the muon $g-2$ from the charm quark using lattice QCD. The calculation is performed on ensembles generated with dynamical (*u*, *d*, *s*) quarks at the SU(3)$_\mathrm{f}$ symmetric point with degenerate pion and kaon masses of around 415 MeV. It includes the connected charm contribution, as well as the leading disconnected Wick contraction, involving the correlation between a charm and a light-quark loop. Cutoff effects turn out to be sizeable, which leads us to use lighter-than-physical charm masses, to employ a broad range of lattice spacings reaching down to 0.039 fm and to perform a combined charm-mass and continuum extrapolation. We use the $\eta _c$ meson to define the physical charm-mass point and obtain a final value of $a_\mu ^\mathrm{HLbL,c}= (2.8\pm 0.5) \times 10^{-11}$, whose uncertainty is dominated by the systematics of the extrapolation. Our result is consistent with the estimate based on a simple charm-quark loop, whilst being free of any perturbative scheme dependence on the charm mass. The mixed charm–light disconnected contraction contributes a small negative amount to the final value.

## Introduction

The anomalous magnetic moment of the muon, $a_\mu \equiv (g-2)_\mu /2$, is one of the most precisely measured quantities in fundamental physics. Currently, the experimental world average [1, 2] and the theoretical evaluation of the 2020 White Paper (WP) [3] based on the Standard Model (SM) of particle physics are in tension at the $4.2\sigma$ level. The theory uncertainties are entirely dominated by the hadronic contributions. Surprisingly, a lattice-QCD based calculation [4] of the leading hadronic contribution finds a larger value than the dispersion-theory based estimate of the WP, which would bring the overall theory prediction into far better agreement with the experimental value of $a_\mu$. Thus it will be vital to resolve the tension between the different determinations of the leading hadronic contribution in order to strengthen the unique test of the SM offered by the anomalous magnetic moment of the muon.

A subleading hadronic contribution to $a_\mu$, the hadronic light-by-light (HLbL) contribution, also contributes sizeably to the error budget of the SM prediction. The HLbL contribution is significantly more complex to evaluate than the leading hadronic contribution; however, because it is suppressed by an additional power of the fine-structure constant $\alpha$, it only needs to be determined at the ten percent level. In the past decade, the HLbL contribution, too, has been evaluated using either dispersive methods [5], assisted by short-distance constraints and hadron structure input [6–18], or lattice QCD. In this case, good agreement is found between the dispersive [3] and the two lattice evaluations [19, 20] within the quoted uncertainties.

One missing ingredient in the otherwise complete HLbL calculation of [20] is the contribution of the charm quark. The present paper addresses this missing contribution. Since the charm quark is much heavier than the muon, on general grounds [21–23] one expects this contribution to be in a regime where it is roughly proportional to $m_\mu ^2/m_{c}^2$. In phenomenological estimates, it has been evaluated using the prediction based on quantum electrodynamics (QED), amended for the appropriate charge and colour factors [24]. We quote the value and uncertainty from the 2020 White Paper (WP) [3],

1 $$\begin{aligned} a_\mu ^\mathrm{HLbL,c}(\mathrm{WP}) = (3 \pm 1) \times 10^{-11}. \end{aligned}$$

While the central value comes from evaluating the QED-like quark-loop, the uncertainty has been estimated conservatively based on the size of the $\eta _c$ pole contribution [12]. Since the WP appeared, the leading radiative correction to a massless quark loop has also been computed [25]. The main goal of this paper is thus to test the prediction (1) using lattice QCD, in case a qualitative effect might have been missed. Certainly, this contribution is small compared to the overall uncertainty $43\times 10^{-11}$ of the WP prediction for $a_\mu$, however the other uncertainties are also expected to shrink, especially if the issues in the leading hadronic contribution can be resolved.

Our second motivation for addressing the charm HLbL contribution from first principles is to answer the qualitative question whether approximating this contribution via a simple quark loop is adequate. In lattice QCD, the calculation involves computing charm propagators on an ensemble of non-perturbative background SU(3) gauge fields. If the simple quark-loop picture is approximately correct, the details of this gauge field should not matter much, and the charm propagators can be replaced by free Dirac propagators. In this case, the sensitivity to the sea quarks enters (at the earliest) at quadratic order in $\alpha _s(m_c)$, the strong coupling constant at the scale of the charm mass. It is largely for this reason that we will focus on the SU(3)$_\mathrm{f}$-symmetric mass point with $m_\pi =m_K\simeq 415$ MeV, enabling us to reach sufficiently fine lattices at a moderate computational cost.

A further aspect of the quark-loop picture is that the various disconnected diagrams entering the HLbL amplitude are expected to be small. In contrast, if the $\eta _c$ pole exchange or *D* meson loops played a sizeable role in the charm-quark contribution, the leading disconnected charm contribution, consisting of a charm loop and a light-quark loop, each attached to two electromagnetic currents, would be sizeable (in analogy to the analyses in [26] and Appendix A of Ref. [20] for the three-flavour case). We recall that for the light quarks, individual mesons, especially the pseudoscalars $\pi ^0,\eta ,\eta '$, contribute substantially to $a_\mu ^\mathrm{HLbL}$, even at the aforementioned SU(3)$_\mathrm{f}$-symmetric point [27]. In lattice QCD, we can quantitatively test the relevance of the disconnected contributions.

This paper is organized as follows. We describe our lattice setup, the tuning of the charm quark mass and our specific representation of $a_\mu ^\mathrm{HLbL}$ in Sect. 2. Section 3 provides some basic theory expectations concerning the connected and leading disconnected contributions involving a charm quark. Section 4 presents our lattice results on the connected contribution for a sequence of increasing charm-quark masses, and Sect. 5 contains our results at the target charm mass for the leading topology of disconnected diagrams. We provide our final result and conclude in Sect. 6. Appendix A describes a test of our methods at a heavy quark mass in lattice QED, while Appendix B contains tables of results for the connected charm contribution on individual ensembles and Appendix C a representative set of fit results.

## Lattice setup

We have performed lattice-QCD calculations on gauge ensembles provided by the Coordinated Lattice Simulations (CLS) initiative [28], which have been generated using three flavours of non-perturbatively O(*a*)-improved Wilson-clover fermions and with the tree-level-improved Lüscher–Weisz gauge action. As in Ref. [27], where we computed the (*u*, *d*, *s*) quark contribution, we consider only ensembles realizing exact SU$(3)_\mathrm{f}$-symmetry. On these ensembles, the mass of the octet of light pseudoscalar mesons is approximately 415 MeV. The parameters of these ensembles, which correspond to six different values of the lattice spacing, are summarized in Table 1.

**Table 1:** The SU$(3)_\mathrm{f}$-symmetric ensembles used in this work. Each ensemble is parametrized by the gauge coupling parameter $\beta \equiv 6/g_0^2$, the (*u*, *d*, *s*)-quark hopping parameter $\kappa$, the lattice size, and the temporal boundary condition. The lattice spacings *a* were determined in Ref. [29], apart from A653 and J500, where the lattice spacings were estimated from the ratio of the Wilson flow parameter $t_0$; the errors on the lattice spacing for these two ensembles (in bold) are simply estimated by scaling of the total error of the neighboring lattice spacings. Their pion masses (marked with asterisk) have been measured independently for this work

Label	$\beta$	$\kappa$	$L^3\times L_T$	Temporal B.Cs	*a* (fm)	$m_{\pi ,K,\eta }$ (MeV)
A653	3.34	0.1365716	$24^3\times 48$	Periodic	0.09930(**122**)	413(5)${}^*$
H101	3.40	0.13675962	$32^3\times 96$	Open	0.08636(98)(40)	418(5)
B450	3.46	0.13689	$32^3\times 64$	Periodic	0.07634(92)(31)	417(5)
N202	3.55	0.137000	$48^3\times 128$	Open	0.06426(74)(17)	412(5)
N300	3.70	0.137000	$48^3\times 128$	Open	0.04981(56)(10)	421(5)
J500	3.85	0.136852	$64^3\times 192$	Open	0.03910(**46**)	413(5)${}^*$

### Calibrating the charm mass and current

The connected contribution to $a_\mu ^\mathrm{HLbL}$ and the two-point correlation function of ${\bar{c}} \gamma _5 c$ were computed on all ensembles of Table 1 for several (5 or 7) values of the charm-quark bare subtracted mass $am_\mathrm{c}=(\kappa _c^{-1}-\kappa _\mathrm{crit}^{-1})/2$, with values of $\kappa _c$ chosen to interpolate between the physical strange and charm hopping parameters. A determination of the latter is available from Ref. [30], obtained by tuning the $D_s$ meson mass to its physical value. For the (dominant) connected contribution however, we choose the physical charm-mass point as the one defined by the physical value of the $\eta _c$ meson mass. When we determine the $\eta _c$ mass, we do not include the disconnected diagram in the two-point function of the charm pseudoscalar density. This procedure corresponds to using the operator ${\bar{c}}' \gamma _5 c$ ($am_\mathrm{c^\prime } = am_\mathrm{c}$), where the degenerate quark flavours *c* and $c'$ are both treated at the partially-quenched level. It should be noted that the tuning of Ref.  [30] by the $D_s$ yields a heavier-than-physical $\eta _c$ meson at our $\text {SU}(3)_\text {f}$-symmetric point. This comes from the quark masses at the latter point being lighter than the physical strange quark, and a $D_s$-tuning *de facto* absorbs this effect into the charm-quark mass [31].

The reason for using lighter-than-physical charm quark masses is that we expect discretisation effects to become more and more significant when the charm mass increases. For a rough estimate of the typical size of discretisation effects, [31] found that the effective speed of light (as defined by the dispersion relation of a meson) for physical-mass charm quarks at worst deviates from unity by 20% in our setup.

The finite renormalisation factor $Z_V^c(g_0,am_\mathrm{c})$ for the local charm current ${\bar{c}} \gamma _\mu c$ was determined by requiring the corresponding charge of the ground-state meson created when $\bar{c}' \gamma _5 c$ acts on the vacuum to be unity. The meson correlators were computed using $Z_2 \times Z_2$ stochastic wall sources [32, 33]. The quark-mass dependence of $Z_V^c(g_0,am_\mathrm{c})$ is quite strong, especially at coarse lattice spacings. Since this factor enters to the third power into our final result, we determine it directly for every one of the bare quark-mass values. This is the same procedure that was implemented for the charm renormalisation in [30].^1^

### Computing the charm contribution to $a_\mu ^\mathrm{HLbL}$

We apply the formalism described and used in [20, 27] and therefore only recall the main aspects. The starting point of our calculation is the master formula^2^

2 $$\begin{aligned} a_\mu ^\mathrm{HLbL}= & {} \int _0^\infty d|y|\; f(|y|),\quad \nonumber \\ f(|y|)= & {} \frac{m_\mu e^6}{3} 2\pi ^2 |y|^3 \int _x \; \bar{\mathcal L}_{[\rho ,\sigma ];\mu \nu \lambda }(x,y)\;i\widehat{\Pi }_{\rho ;\mu \nu \lambda \sigma }(x,y).\nonumber \\ \end{aligned}$$

**Fig. 1:**
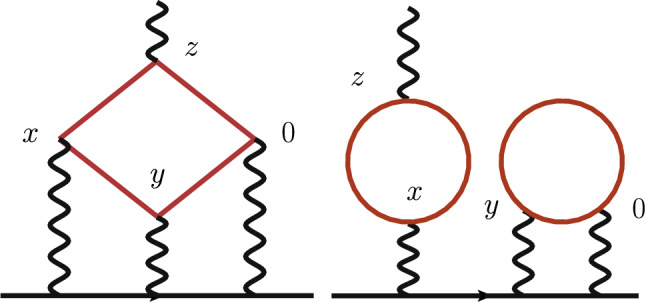
The fully connected charm contribution (left) and the (2+2) Wick contraction (right, with at least one loop corresponding to a charm quark) are the two Wick contractions computed in this work

Here $e^2/(4\pi )=\alpha _\mathrm{QED}$ is the fine-structure constant and $m_\mu$ the muon mass. The QED kernel $\bar{\mathcal L}$ has been computed in the continuum [34] and represents the contributions of the photon and muon propagators and vertices in the diagrams of Fig. 1. There is a lot of freedom to alter the kernel without changing $a_\mu ^\mathrm{HLbL}$ in the continuum and in infinite volume. Specifically, we use the kernel $\bar{\mathcal L}^{(\Lambda )}$ defined in [27] with $\Lambda =0.40$ throughout.^3^ The tensor $i\widehat{\Pi }$ is a Euclidean hadronic four-point function with one of its vertices weighted linearly in one of its coordinates,

3 $$\begin{aligned} \begin{aligned} i\widehat{\Pi }_{\rho ;\mu \nu \lambda \sigma }( x, y)&= -\int _z z_\rho \, \Big \langle \,j_\mu (x)\,j_\nu (y)\,j_\sigma (z)\, j_\lambda (0)\Big \rangle _\mathrm{QCD}. \end{aligned}\nonumber \\ \end{aligned}$$

The field $j_\mu (x)$ appearing above is the hadronic component of the electromagnetic current,

4 $$\begin{aligned} j_\mu (x) = \sum _f {\mathcal Q}_f \;({\bar{q}}_f \gamma _{\mu } q_f)(x). \end{aligned}$$

Here we focus on the contributions involving the charm current, $\bar{c}\gamma _\mu c$. The QCD four-point function receives contributions from five classes of Wick contractions. First, we will focus on the fully-connected charm contribution, which involves four charm currents; for this contribution, we apply Eq. (7) of Ref. [20] with the flavour index set to charm, $j:=c$. Second, we will consider the disconnected contributions involving two quark loops, each of which containing two vector vertices, with either one or both loops consisting of charm propagators. Here we apply Eq. (11) of Ref. [20] with the flavour indices *i*, *j* running over $\{u,d,s,c\}$ under the constraint that at least one of them take the value *c*. The connected and (leading) disconnected contributions are illustrated in Fig. 1.

## Theory expectations

The simplest prediction for the light-by-light contribution of a heavy ‘charm’ quark to $10^{11} a_\mu$ relies on the analytic QED result originally applied to the $\tau$ lepton loop [35, 36]. Taking into account the colour factor $N_c=3$ and the charge factor $(2/3)^4$, it is given by the function

5 $$\begin{aligned} h(m_Q)= & {} 5.10382 \frac{1}{{\hat{m}}_Q^2} + \Big (-0.176225 -0.0567645\log ({\hat{m}}_Q^2) \nonumber \\&-0.00459931 \log ^2({\hat{m}}_Q^2) \Big ) \frac{1}{{\hat{m}}_Q^4}, \end{aligned}$$

with ${\hat{m}}_Q$ is the heavy-quark mass in GeV. Already by $m_Q=0.75$ GeV, the O$(m_Q^{-4})$ terms only represent a reduction of the leading term by five percent. These terms certainly represent a small correction for $m_Q$ around the physical charm mass. Here we have dropped known higher-order terms in $1/m_Q$. We will take the function $h(m_Q)$ as a baseline for comparison with our lattice results for the fully connected charm contribution.

For the (2+2) disconnected contribution involving one charm and one light-quark loop, it is less straightforward to make a ‘baseline’ prediction. The scalar-QED prediction for the contribution to $a_\mu ^\mathrm{HLbL}$ of the $D^\pm$ meson loop is $-0.33\times 10^{-11}$ [36], to be roughly doubled in order to include the $D_s$ loop. Taking into account the charge factor of $2\cdot 3\cdot {\mathcal Q}_c^2({\mathcal Q}_u^2+{\mathcal Q}_d^2+{\mathcal Q}_s^2)=\frac{144}{81}$ relevant for the charm–light (2+2) contribution (see [20], Appendix A^4^), one arrives at the prediction of $a_\mu ^{2+2:lc}= -0.58\times 10^{-11}$ when treating the $D^+, D^0,D_s$ meson loops within scalar QED.^5^ The absolute value of this prediction is surely an overestimate, given that electromagnetic form factors of the *D* mesons should suppress this prediction substantially: in the case of the pion loop, the suppression factor is almost three, and for the kaon almost ten [3, 36]. All in all, these considerations finally lead us to expect an order of magnitude of $(-0.3\pm 0.3)\times 10^{-11}$.

In addition to the short-distance effect estimated above, the charm–light disconnected diagrams also involve a longer-distance contribution, whose size it is useful to estimate by theory arguments, given the difficulty of measuring the correlation function in the infrared. The intuitive idea is that the heavy-quark loop shrinks almost to a point in coordinate-space,^6^ acting effectively like a local gauge-invariant gluonic operator from the point of view of the ‘low-energy effective theory’, which is QCD with (*u*, *d*, *s*) quarks. This picture can be formalized by writing an effective Lagrangian for the effective coupling induced between two photons and gluonic fields, much as in the classic work of Euler and Heisenberg [37]. This effective Lagrangian ${\mathcal L}^{(c)}_{2\gamma 2g}$ has been calculated long ago [38]; each term of the Lagrangian contains two photonic and two gluonic field strength tensors. From here, one infers the operator equation

6 $$\begin{aligned}&{\mathcal Q}_c^2\;\mathrm{T}\{ ({\bar{c}}\gamma ^\mu c)(x)\; ({\bar{c}}\gamma ^\mu c)(y)\} \nonumber \\&\quad = \frac{1}{e^2}\frac{\delta ^2}{\delta A_\mu (x) \delta A_\nu (y)} \int d^4w\; {\mathcal L}^{(c)}_{2\gamma 2g}(w), \end{aligned}$$

$A_\mu$ being the photon field, which shows that the charm loop acts at low energies like a set of gluonic operators such as $\alpha _s G_{\mu \nu }^a G_{\mu \nu }^a$ or $\alpha _s G_{\mu \nu }^a \tilde{G}_{\mu \nu }^a$. The main observation is that, on dimensional grounds, the effective Lagrangian is overall multiplied by a $1/m_c^4$ factor, indicating a strong suppression.

The argument above shows that a light flavour-singlet meson such as the scalar $f_0$ or the pseudoscalar $\eta '$ can propagate between the charm loop and the light-quark loop, albeit with a very suppressed coupling to the charm loop. To get an estimate of this contribution, which is long-range in comparison to the length-scale $(2m_c)^{-1}$, we use Eq. (6) to find out roughly how much the charm part of the electromagnetic current by itself contributes to the $\eta '$ transition form factor (TFF). Note that this contribution is independent of the photon virtualities, as long as these are small. Using the estimate $\langle 0|\alpha _s G \tilde{G} | \eta '\rangle \approx 0.5\,\mathrm{GeV}^3$ based on Ref. [39], while the TFF normalisation amounts to $|{\mathcal F}_{\eta '\gamma \gamma }(0,0)| \simeq 0.34\, \mathrm{GeV}^{-1}$ (see for instance [40]), we obtain a contribution of about $8\times 10^{-4}\,\mathrm{GeV}^{-1}$ to ${\mathcal F}_{\eta '\gamma \gamma }$ from the charm current. Since the $\eta '$ exchange contributes about $14.5\times 10^{-11}$ to $a_\mu ^\mathrm{HLbL}$ [3], proportionally to its TFF at each end of $\eta '$ propagator, we arrive at the order-of-magnitude estimate of $0.01\times 10^{-11}$ for the contribution to $a_\mu ^\mathrm{HLbL}$ of the $\eta '$ in the (2+2) charm–light diagrams. Even with a potential logarithmic enhancement [41], this is much smaller than our final uncertainty and cannot presently be resolved in our lattice calculations.

**Fig. 2:**
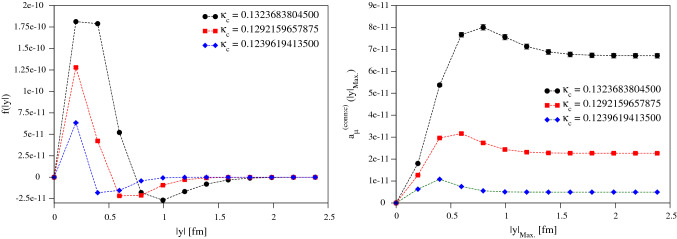
The local-conserved integrands (left) and partially-integrated results (right) for a selection of $\kappa _c$s (heaviest, middle, and lightest $aM_{\eta _c}$) for our coarsest ensemble A653. Dashed lines are to guide the eye

In addition to the Wick-contraction topologies considered above, the (3+1) topology with the single-current loop consisting of a charm propagator deserves some attention, since this contribution is neither SU(3)$_\mathrm{f}$ nor $1/N_c$ suppressed relative to the (2+2) topology, $N_c$ being the number of colours. In perturbation theory, the (3+1) contribution starts at O($\alpha _s^3$) rather than at O($\alpha _s^2$), while involving the same minimal number of charm propagators. Furthermore, the quark-charge and multiplicity factors numerically suppresses this contribution by a relative factor of three^7^ since it is weighted by $4\cdot ({\mathcal Q}_u^3+{\mathcal Q}_d^3+{\mathcal Q}_s^3){\mathcal Q}_c = 48/81$, while the charm–light (2+2) diagrams are weighted by 144/81, as noted above. A factor of three suppression relative to the (2+2) charm–light contribution is thus expected.

## Lattice results for the connected contribution

As a way of validating our computational methods, our analysis has been guided by a lepton-loop calculation, much like in Ref. [27]: in Appendix A we investigate the applicability of our QED-kernel implementation at particularly heavy scales by comparing the lepton-loop contribution to $a_\mu ^\mathrm{LbL}$ to the known analytical expression [35]. While the agreement is acceptable at fairly heavy lepton mass, the study suggests that cut-off effects will be significant and working at unphysically-light charm mass might allow for a better handle on these effects. The physical charm mass will therefore be approached via a simultaneous extrapolation in the quark mass and in the lattice spacing.

**Fig. 3:**
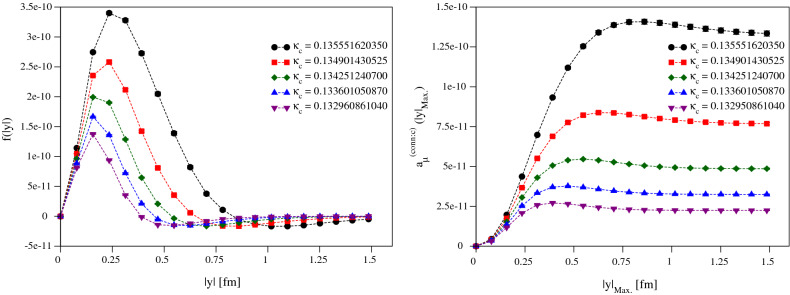
The local-conserved integrands (left) and partially-integrated results (right) for all of our $\kappa _c$s, from our finest ensemble J500

### Results at individual quark masses

For the connected part of $a_\mu ^\mathrm{HLbL,c}$, we have performed computations with the vector current connected to the external on-shell photon (the *z*-vertex in Eq. (3)) being either symmetrised-conserved^8^ or local, while the rest of the currents are kept local. For each ensemble, we have tuned $\kappa _c$ to get five to seven different $\eta _c$-masses, ranging from around 1.3 to 2.6 GeV. In order to better control rotational-symmetry breaking effects (and keep the higher-order lattice artifact coefficients the same) we will only use *f*(|*y*|) along the lattice direction (1,1,1,1) for all ensembles.

Figure 2 shows an example of our data for the A653 ensemble. The integrand is steeply-peaked at short distances and becomes more so at heavier quark masses (smaller $\kappa _c$). As can be seen from the partially-integrated results, even the lightest charm-quark-mass lattice data used here completely saturates the integral and therefore there is no need to perform any tail-extension procedure, and just the lattice (trapezoid-rule) integral suffices. We also note that the overall integrand and integral becomes substantially smaller as $\kappa _c$ decreases, representing the fact that this integral must vanish in the limit $\kappa _c\rightarrow 0$. There is a strong negative tail in the integrand causing a fairly significant cancellation for the overall integral, which becomes smaller as the charm-mass decreases. At very low $\kappa _c$ on coarse lattices it is unlikely that we will be able to properly resolve the peak of the integrand and end up with a lower estimate due to the negative tail cancelling against the peak contribution more than it should. As we move to finer lattices and the resolution at low |*y*| improves, we resolve the peak structure much better, as illustrated in Fig. 3.

### Mass-dependence of the connected contribution

**Fig. 4:**
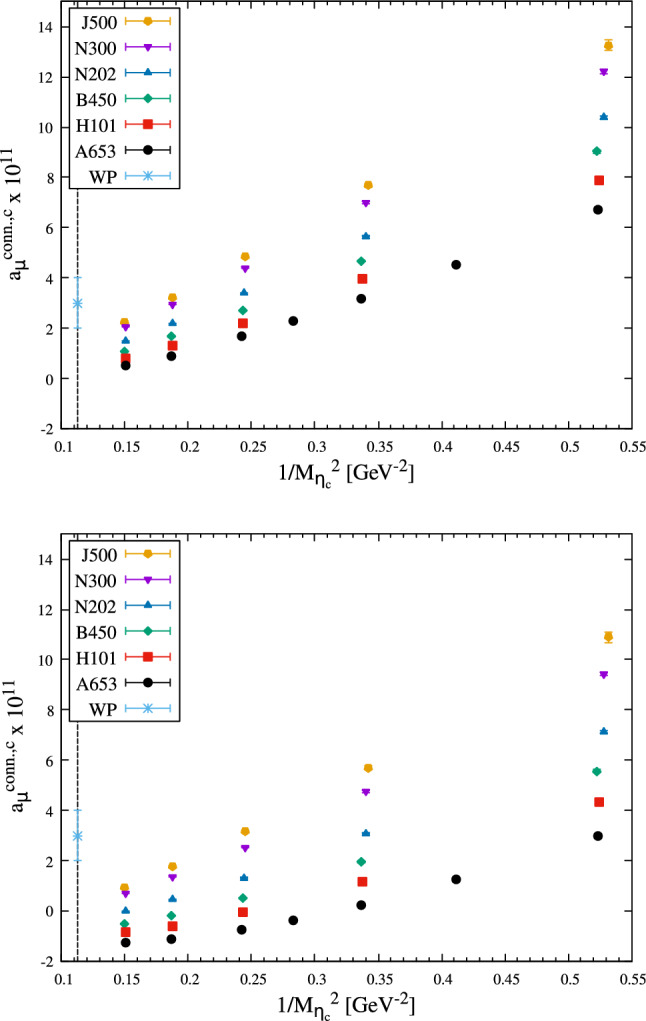
Lattice results for the connected part of $a_\mu ^\mathrm{HLbL}$ in units of $10^{11}$ for the local-conserved (top) and local-local (bottom) data (see text). The black vertical line on the left indicates the physical value of $1/M_{\eta _c}^2$. The light blue point lying at physical $1/M_{\eta _c}^2$ is the estimate from Ref. [3]

The results are given in Tables 3, 4, 5, 6, 7 and 8 of Appendix B and summarized in Fig. 4.

Expectations are that $a_\mu$ scales with $m_\mu ^2/m_\mathrm{{heavy}}^2$ [21–23], so it is instructive to focus on the dependence of $a_\mu ^\mathrm{HLbL,c}$ on $1/M_{\eta _c}^2$. The data show a clear monotonic decrease as $1/M_{\eta _c}^2$ is decreased toward its physical value, starting (for the lightest charm quarks) at or above the WP prediction and ending (for the heaviest charm quarks) at or below the WP value. At similar $\eta _c$ mass, the data have a large spread between the coarsest and finest ensemble, indicating strong discretisation effects.

At this point, it is useful to compare the two choices of discretisations for the currents: the spread is larger in the local-local data than in the local-conserved data. Furthermore, the curvature in $1/M_{\eta _c}^2$ has a stronger dependence on the lattice spacing in the local-local data. In addition, the fact that the coarse local-local data at large $M_{\eta _c}$ become negative makes it more difficult to describe the data using a fit ansatz. For these reasons, we decide to base our determination of $a_\mu ^\mathrm{HLbL,c}$ solely on the analysis of the local-conserved data.

### Extrapolation to the continuum and to the physical charm mass

Due to the heaviness of the valence charm quark, the intermediate states that could potentially contribute to the correlation function in question should be much suppressed at large distances; see the discussion in Sect. 3. Indeed, this can be seen by the saturation of the tail of the lattice integrand (Figs. 2, 3). For this reason, in the approach to the physical point, we assume that the finite-size effects are minor and only extrapolate in the $\eta _c$-meson mass and lattice spacing *a*. The statistical error on each individual data point is at the percent-level, which is comparable to the quoted error on the lattice spacings given in Table 1; therefore, it is crucial to include the error on the lattice spacing while performing an extrapolation to the physical point.

To this end, a global fit is performed based on a Bayesian approach [43], where we promote each lattice spacing to a fit-parameter and associate to it a Gaussian prior with the central value and the width taken to be the quoted central value of the lattice spacing $\bar{a}$ and its error $\Delta a$ respectively. Although the parameter space is small, constructing a fit-ansatz with a $\chi ^2/\text {dof}$ on the order of unity is in fact not an easy task. After various attempts, we have identified two classes of ansätze which are able to describe the data with reasonably good $\chi ^2/\text {dof}$.

The most restrictive constraint that we deem important to fulfill is the $m_\mu ^2/m_\mathrm{{heavy}}^2$ scaling of $a_\mu ^\mathrm{HLbL,c}$ in the presence of a heavy scale [21–23]. It is natural to first consider the $\eta _c$-meson mass for such a scale. A challenging part of the construction of fit-ansätze is to handle the apparent non-linear behavior in $1/M_{\eta _c}^2$ of the data (see Fig. 4), which gradually gets milder as we go down in the lattice spacing. This motivates our first class of ansätze, the *P-class*, which consist of linear combinations of a leading term in $1/M_{\eta _c}^2$ and terms in $a^n f(M_{\eta _c})$ with $n\in \mathbb {N}^*$ and *f* an elementary function, treating the non-linearity in $1/M_{\eta _c}^2$ as a lattice artifact.

Another way to account for the $m_\mu ^2/m_\mathrm{{heavy}}^2$ scaling is to use the charm quark mass as a heavy scale. A rough estimate in the non-relativistic limit is that the $\eta _c$-meson mass should be equal to twice the charm quark mass, up to small relative corrections. Based on this observation, we define the *R-class* of fit ansätze, consisting of rational functions:

7 $$\begin{aligned} \frac{P(a,M_{\eta _c})}{\frac{1}{4}(C+M_{\eta _c})^2 + Q(a,M_{\eta _c})}, \end{aligned}$$

where *P* and *Q* are polynomials in both *a* and $M_{\eta _c}$ and *C* is a constant. In principle, *C* can also have non-trivial dependence on *a* and on $M_{\eta _c}$; however, introducing additional parameters to describe this dependence turns out to be unnecessary, as the non-linearity of the data can already be well captured with the form in Eq. (7).

With the aforementioned two fit-ansatz classes, it remains nevertheless difficult to get reasonable $\chi ^2/\text {dof}$ with the whole available dataset. In fact, this is not very surprising, as the resolution of the peak of the integrand becomes worse as $\kappa _c$ and *a* become small (see Fig. 2). Therefore, it is necessary to allow for various cuts to the data. At the same time, as we would like to reach as heavy as possible $M_{\eta _c}$ masses in order to have a better control over the extrapolation to its physical value, it is preferable to discard as few data points as possible. A lattice study in the pure QED case presented in App. A shows that our setup should be valid up to a charm-quark mass of at least 20/3 times that of the muon, with well-controlled cut-off effects. Based on the latter and with a simple linear relation between the physical $\eta _c$ mass and the $\overline{{MS}}$-mass of the charm quark [44], we demand an admissible fit to be able to cover the data points in the range of $1/M_{\eta _c}^2>0.20$ GeV${}^{-2}$.

**Fig. 5:**
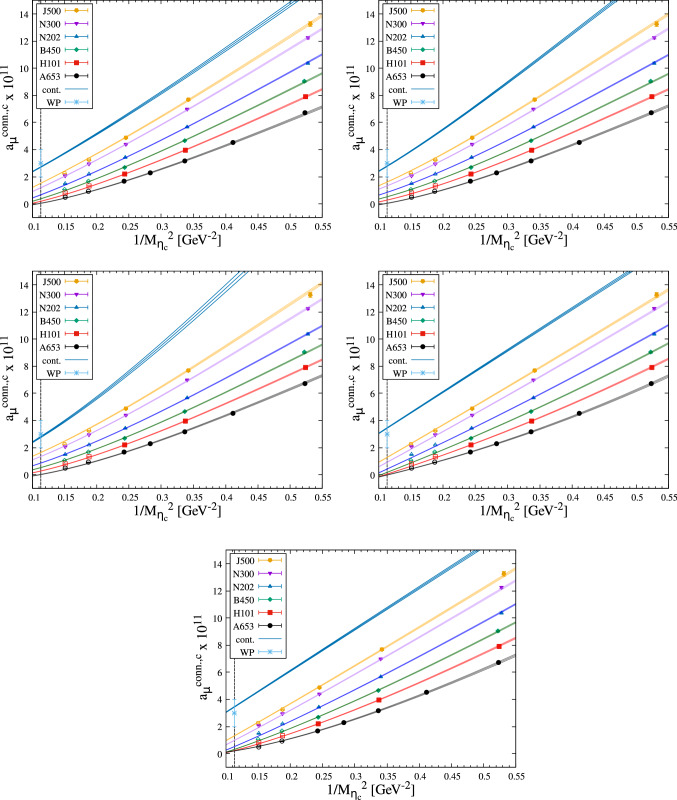
Selected fit results with the dataset D1 (datapoints indicated by filled symbols): fit 1, fit 2, fit 3, fit 4, and fit 5 (left to right from the top to the bottom). The continuum limit result is given by the top-most curve (“cont.”). For other information, see Fig. 4

Our fitting strategy goes as follows: We build fit ansätze from either the P- or the R-class as explained earlier. To avoid overfitting, the number of fit parameters is limited to five. Apart from terms in $a^n M^m$, we have also tried logarithmic terms in *a* or *M* in order to allow for different types of curvature. The four datasets we consider are (D2, D3, and D4 are defined as D1 with extra omissions):

- D1: All ensembles and data points where $1/M_{\eta _c}^2>0.20 \text { GeV}^{-2}$.
- D2: D1 where data from the coarsest lattice (A653) are omitted.
- D3: D1 where the lightest $M_{\eta _c}$ data of each ensemble are omitted.
- D4: D1 where $aM_{\eta _c}> 0.8$ are omitted.

Given that our final uncertainty estimate is dominated by systematics due to the choice of fit ansatz and that attempts at correlated fits yielded a poor fit quality, we choose to neglect correlations between different $M_{\eta _c}$ on the same ensemble. Although this harms the statistical interpretation of $\chi ^2$ and *p*-values computed in the standard way, we nevertheless use these to judge relative fit quality. Our criterion for an admissible fit is one with a *p*-value between 0.05 and 0.95, for which the extrapolated $a_\mu$ and the *p*-value are stable under variation of the dataset choice.

We have tested various fit ansätze from both the P- and R-classes and found that the following five-parameter fits are able to describe our data with the quality requirements fulfilled (see Table 9 in Appendix C and Fig. 5):

8 $$\begin{aligned} \begin{aligned} \text {Fit 1:}\quad a_\mu (a,M_{\eta _c})&= \frac{A+BaM_{\eta _c}^2}{\frac{1}{4}(C+M_{\eta _c})^2 + (D+Ea^2M_{\eta _c}^2)^2},\\ \text {Fit 2:}\quad a_\mu (a,M_{\eta _c})&= \frac{A+BaM_{\eta _c}}{\frac{1}{4}(C+M_{\eta _c})^2+(D+Ea^2M_{\eta _c}^2)^2},\\ \text {Fit 3:}\quad a_\mu (a,M_{\eta _c})&= \frac{A+Ba^2M_{\eta _c}^2}{\frac{1}{4}(C+M_{\eta _c})^2+(D+EaM_{\eta _c}^2)^2},\\ \text {Fit 4:}\quad a_\mu (a,M_{\eta _c})&= Aa + \frac{B+Ca^2}{M_{\eta _c}^2} + Da^2 + E\frac{a^2}{M_{\eta _c}^4},\\ \text {Fit 5:}\quad a_\mu (a,M_{\eta _c})&= Aa + \frac{B+Ca^2}{M_{\eta _c}^2} + Da^2 + E\frac{a^2}{M_{\eta _c}^2}\ln M_{\eta _c}. \end{aligned}\nonumber \\ \end{aligned}$$

A further important feature of these fits is that they qualitatively follow the trend of the data even in the region $1/M_{\eta _c}^2 < 0.2$ GeV${}^{-2}$.

As a general feature, the P-class ansätze tend to lead to larger results for $a_\mu (0,M_{\eta _c}^{\text {Phys}})$ as compared to the R-class. As there is no exclusive theoretical argument for the finite-lattice-spacing behaviour of these functions, and our data seem not to be able to unambiguously rule out any of these classes, our decision is to include the fit results with good $\chi ^2/\text {dof}$ from both of them (see Table 9). More specifically, our final result is the average of our lowest (Fit 1, D3 : 2.64(4)) and our largest (Fit 5, D2 : 3.47(3)) values and we assign a generous systematic error estimate by quoting half the difference of the two, which brings us to our estimate for the connected contribution,

9 $$\begin{aligned} a_\mu ^\mathrm{HLbL,c}{}^{\text {,conn.}} = 3.1(4)\times 10^{-11}. \end{aligned}$$

Our error on this quantity is entirely dominated by the systematic error from our modeling of its dependence on *a* and $M_{\eta _c}$.

### Comparison to the QED-based prediction

**Fig. 6:**
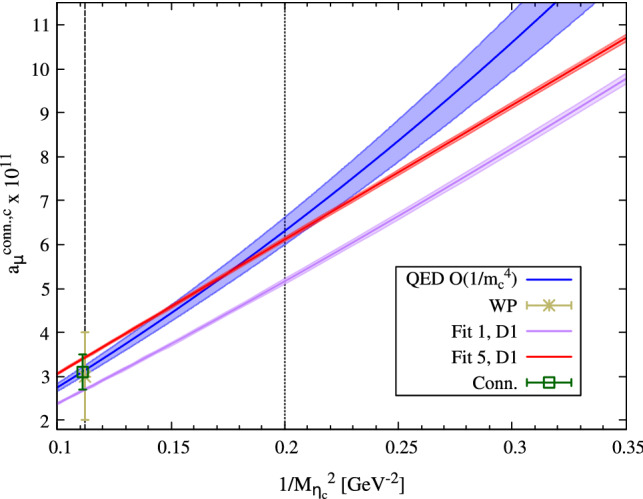
The $\eta _c$-mass-dependence of $a_\mu ^\mathrm{conn.,c}$: comparison between our continuum-extrapolated results using Fit 1 (lower, in magenta) and Fit 5 (higher, in red) based on dataset D1 and the QED-based prediction (in blue). The band on the latter indicates a $\pm 5\%$ change in the mass-shift relating the charm-quark mass to the $\eta _c$ mass (see text). The dotted vertical line indicates the upper bound for the $\eta _c$ mass included in the fits. Our estimate for the connected contribution, Eq. (9), is marked with ‘Conn.’ (a horizontal offset is applied for visibility)

To close the study of the connected contribution, we compare our result for $a_\mu ^\mathrm{HLbL,c}{}^{\text {,conn.}}$ to the charm-quark loop evaluated analytically within QED as given in Eq. (5). To make contact with that expression, we need to specify the relationship between the $\eta _c$ mass and the charm-quark mass. As explained while discussing the R-class of fit-ansätze, we assume the $\eta _c$ mass to be twice the charm quark mass plus an almost charm-quark-mass independent mass-shift within a given window of $M_{\eta _c}$. We estimate the mass-shift using the $\overline{{MS}}$ charm-quark mass and the physical $M_{\eta _c}$ and assign a five-percent uncertainty to this quantity. The prediction from this prescription is displayed in Fig. 6 together with our fit results. The difference between truncating at O$(1/m_c^2)$ and at O$(1/m_c^4)$ is tiny compared to the uncertainty that we assign to the mass-shift inferred from our prescription. It is worth noting that, even though the QED-based prediction gives a result that falls in the bulk of our estimate Eq. (9) at the physical charm mass, the milder curvatures in $1/M_{\eta _c}^2$ of the representative fit results suggest that non-perturbative effects are still significant at intermediate masses.

## The disconnected contribution

The disconnected parts of the charm contribution are expected to be very small. From the outset, we neglect the (3+1), (2+1+1), and (1+1+1+1) Wick-contraction topologies, based partly on them being consistent with zero for the light quark contribution, as found in [20], and partly on the arguments laid out in Sect. 3. This leaves us with the (2+2) topology, which is a sizeable contribution in the light-quark $a_\mu ^\mathrm{HLbL}$ result. This contribution can be broken into the mixed charm–light and the charm–charm contributions, with the former (by analogy with the strange sector) expected to be the major contribution.

As the disconnected contribution is still an expensive calculation, we have limited ourselves to a single charm-quark mass determined by $\kappa _c$ from the $D_s$-tuning of Ref. [30]. This tuning is suboptimal for our present purposes, an aspect we return to below. We will also use the $Z_V^{c}$ values from [30], except for ensemble A653, where we computed the renormalisation factor ourselves. We employ exclusively local vector currents and restrict ourselves to the ensembles N300, N202, B450, and A653, reusing data for the light-quark loop from Ref. [27].

**Fig. 7:**
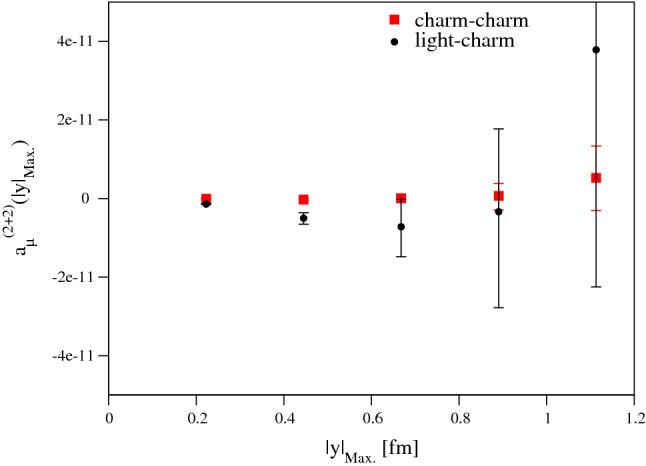
The (2+2) charm–light and charm–charm partially-integrated data for ensemble N202

**Table 2:** The charm–light and charm–charm (2+2) contributions to $a_\mu ^\mathrm{HLbL}$

Ensemble	$\kappa _c$	$Z_V^c$	$a_\mu ^{2+2:lc}\times 10^{11}$	$a_\mu ^{2+2:cc}\times 10^{11}$
A653	0.119759	1.32265	$-3.24$(99)	$-0.06$(2)
B450	0.125095	1.12972	$-0.53$(27)	$+0.01$(2)
N202	0.127579	1.04843	$-0.48$(14)	$-0.03$(2)
N300	0.130099	0.97722	$-0.39$(8)	$-0.03$(1)

A plot of the partially-integrated charm–light and charm–charm disconnected contributions for ensemble N202 is shown in Fig. 7. It is clear that both of these contributions are noisy, small, negative, and very short-distance. Again this means we can use the lattice integral directly for our final result, and we use a simple constant fit to the partially-integrated result for our final determination. Based on the numerical evidence from Figs. 2, 3 that the connected contribution becomes very short-ranged as the charm mass is increased towards its physical value, as well as the theoretical arguments of Sect. 3, we start this fit between 0.4 and 0.5 fm. Table 2 shows our results for this procedure and we see that A653 is an extreme outlier in the charm–light contribution. The other, finer, ensembles yield values much smaller and consistent with one another. We decide to omit this coarse ensemble entirely and fit the remaining charm–light data to a straight line in the variable $a^2$. This leads to the result $a_\mu ^{\text {HLbL,c,(2+2)}} = -0.28(21)\times 10^{-11}$.

We now come back to the issue of the tuning of the charm quark mass. The CLS ensembles we are using are designed to have the trace of the quark mass matrix equal to its physical value, to a rather good approximation [29]. We remind the reader that for the connected contribution, we chose the charm-quark mass such that the physical $\eta _c$ mass is reproduced. With this choice, the dependence of charm correlators on the SU(3)$_\mathrm{f}$ breaking parameter $[m_s-(m_u+m_d)/2]$ is expected to be small, being a pure sea quark effect. As a consequence, the extrapolation to physical (*u*, *d*, *s*) quark masses is expected to be very mild. This is not the case if we tune the mass ${\bar{M}}_D$ of the triplet of *D* mesons at our SU(3)$_\mathrm{f}$-symmetric point to the physical $D_s$ meson, $M_{D_s}=1.968$ GeV. By contrast, if we tune ${\bar{M}}_D$ to the average $({\bar{M}}_D)_\mathrm{phys} \equiv \frac{1}{3}[M_{D^+}+M_{D^0}+M_{D_s}]_\mathrm{phys}=1.901$ GeV of the physical *D* meson masses, then we again avoid a valence-quark effect in the approach to physical quark masses. It is also interesting to ask, how different a tuning this represents as compared to the tuning via the $\eta _c$ mesons mass. We have found that the $\eta _c$ meson mass, extrapolated to the charm mass where ${\bar{M}}_D= ({\bar{M}}_D)_\mathrm{phys}$, amounts to 2.97(4) GeV, which is consistent with its physical value. This is an indication that sea quark effects are indeed small in the charm sector.

These observations lead us to apply a small correction to the charm–light disconnected contribution, to bring it to the point where ${\bar{M}}_D$ takes the value $({\bar{M}}_D)_\mathrm{phys}$. Assuming that the disconnected contribution is roughly proportional to $1/{\bar{M}}_D^2$, we multiply our continuum-extrapolated result obtained at ${\bar{M}}_D = M_{D_s}$ with the ratio $(M_{D_s}/{\bar{M}}_D)_\mathrm{phys}^2$, leading to the final result

10 $$\begin{aligned} a_\mu ^{\text {HLbL,c,(2+2)}} = -0.30(23)\times 10^{-11}. \end{aligned}$$

We neglect the charm–charm contribution as its contribution is far smaller than our final error for the charm–light.

## Discussion of the results and conclusion

We have determined the charm-quark contribution to hadronic light-by-light scattering in the anomalous magnetic moment of the muon. We find that the lattice determination of this quantity is challenging, specifically in the modeling of the connected contribution’s discretisation effects: the associated systematic error dominates our final error budget. As expected from the charm-loop picture, the connected contribution turns out to be the most significant overall. We find the charm–light disconnected contribution to be negative and much smaller in magnitude than the fully-connected contribution, amounting to a $10\%$ correction with a large uncertainty. The charm–charm disconnected contribution is entirely negligible and we expect all higher-order contributions to be equally insignificant.

Before quoting our final result for the charm contribution to $a_\mu ^\mathrm{HLbL}$, we address the question of its dependence on the (*u*, *d*, *s*) quark masses. The fact that several aspects of our lattice results can be understood via the the charm-quark loop picture is an indication that this dependence must be modest, and we may attempt to estimate its order of magnitude via the ambiguity induced by the choice of the charm-quark tuning condition away from the physical (*u*, *d*, *s*) quark-mass point. We saw in Sect. 5 that tuning the average $D^+$, $D^0$ and $D_s$ mass to its physical value was equivalent, within our uncertainties, to tuning the $\eta _c$ mass to its physical value. Still, we estimate that the connected contribution would potentially be modified by 2% had we chosen the alternative tuning. Another estimate can be based on the idea that the charm contribution is proportional to the sum of the inverses of the charged *D*-meson squared masses. That sum differs again by about two percent from the square inverse of the average $D^+$, $D^0$ and $D_s$ mass. This argument suggests an absolute systematic error of $0.06\times 10^{-11}$, which we conservatively inflate to $0.12\times 10^{-11}$ and add in quadrature to the other uncertainties below. This estimate also covers the (*u*, *d*, *s*) valence-quark mass dependence present in the charm–light disconnected contribution: as discussed in Sect.  3, for that topology we expect a short-range contribution (with a reach of order $(2m_c)^{-1}$), plus a longer-range contribution with a very suppressed amplitude coming from the exchange of (*u*, *d*, *s*) isoscalar mesons. The short-range part is again expected to depend on the light-quark masses mainly via the *D* meson masses as estimated above, while the longer-ranger part is simply too small to affect our estimate.

Our full result from adding Eqs. (9) and (10) together and adding errors in quadrature is

11 $$\begin{aligned} a_\mu ^\mathrm{HLbL,c}= (2.8\pm 0.5)\times 10^{-11}. \end{aligned}$$

This result is completely consistent with the 2020 White Paper estimate of $(3\pm 1)\times 10^{-11}$ [3], and has half its uncertainty.

Combining Eq. (11) with our previous result from the light and strange contributions of $a_\mu ^{\text {HLbL,ls}}=(106.8\pm 15.9)\times 10^{-11}$ [20] obtained with dynamical (*u*, *d*, *s*) quarks yields a fully non-perturbative determination of $a_\mu ^\mathrm{HLbL}$, including all relevant contributions. A last effect not yet accounted for is the charm sea-quark effect on the light-quark contributions, as for instance the $D^+$ meson loop can contribute to the connected four-point function of the down quark in a calculation with dynamical (*u*, *d*, *s*, *c*) quarks. Within a scalar-QED treatment of the *D* meson, we have however estimated this effect to be below $0.1\times 10^{-11}$. Therefore we neglect the charm sea quark effects and arrive at

12 $$\begin{aligned} a_\mu ^\mathrm{HLbL}=(109.6\pm 15.9)\times 10^{-11}. \end{aligned}$$

This concludes our first-generation calculation of hadronic light-by-light scattering in the muon $(g-2)$.

## Figures and Tables

**Fig. 8:**
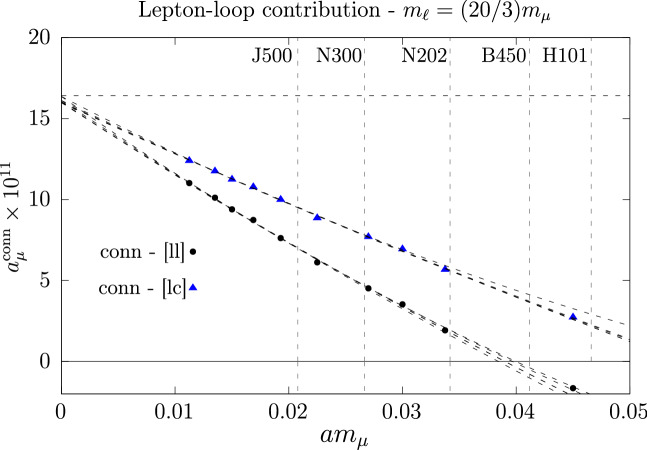
Continuum extrapolation for the lepton loop with $m_\ell /m_\mu = 20/3$, computed on the lattice with two discretisations of the vector current (local or conserved) at the external vertex *z*

## Data Availability

This manuscript has no associated data or the data will not be deposited. [Authors’ comment: The paper itself contains many intermediate results collected in tables.].

## Appendix A: Methodology test for a heavy lepton

With our implementation of the QED coordinate-space kernel, we have been able to reproduce various known light-by-light contributions in the continuum [27, 47, 48] at the one-percent level. The tests performed so far concern physics involving particles with masses on the same order as the muon mass. As our implementation of the QED-kernel relies on interpolating weight functions that are precomputed on a grid [34], it is important for the goal of this paper to test how robust this implementation is for computing contributions from more massive particles (Fig. 8).

As an example of a calculation performed entirely in the continuum, we quote the result we obtain with the kernel $\bar{\mathcal L}^{(\Lambda =0.40)}$ and ‘method 2’ for the lepton-loop contribution with $m_\ell /m_\mu = 4$, namely $a_\mu ^\mathrm{HLbL}=(42.1\pm 0.5)\times 10^{-11}$; the exact result is $(43.175...)\times 10^{-11}$. While this precision is sufficient for our present purposes, it is clear that, using continuum propagators for the lepton loop, the quality and stability of the coordinate-space results degrade when the lepton mass increases.^9^

**Table 3:** Results for ensemble A653, $a_\mu$ has been multiplied by $10^{11}$

$\kappa _c$	$M_{\eta _c}$ [GeV]	$Z_V^{(c)}$	$a_\mu ^{\text {(Conn.[lc])}}$	$a_\mu ^{\text {(Conn.[ll])}}$
0.1239619413500	2.5780	1.15128(7)	0.494(9)	$-1.246$(26)
0.1260635511250	2.3144	1.06998(7)	0.909(13)	$-1.110$(33)
0.1281651609000	2.0316	0.99145(6)	1.657(23)	$-0.726$(42)
0.1292159657875	1.8815	0.95307(6)	2.268(31)	$-0.359$(48)
0.1302667706750	1.7243	0.91530(6)	3.163(44)	0.238(58)
0.1313175755625	1.5589	0.87813(6)	4.527(61)	1.236(73)
0.1323683804500	1.3827	0.84154(9)	6.717(106)	2.973(101)

**Table 4:** Same as Table 3 but for ensemble H101

$\kappa _c$	$M_{\eta _c}$ [GeV]	$Z_V^{(c)}$	$a_\mu ^{\text {(Conn.[lc])}}$	$a_\mu ^{\text {(Conn.[ll])}}$
0.1263626550	2.5764	1.07159(7)	0.811(7)	$-0.827$(15)
0.1280954825	2.3112	1.00793(7)	1.313(10)	$-0.588$(19)
0.1298283100	2.0282	0.94610(6)	2.205(16)	$-0.059$(25)
0.1315611375	1.7212	0.88580(7)	3.952(21)	1.168(36)
0.1332939650	1.3811	0.82708(6)	7.901(69)	4.328(70)

**Table 5:** Same as Table 3 but for ensemble B450

$\kappa _c$	$M_{\eta _c}$ [GeV]	$Z_V^{(c)}$	$a_\mu ^{\text {(Conn.[lc])}}$	$a_\mu ^{\text {(Conn.[ll])}}$
0.128043750	2.5817	1.02163(4)	1.073(7)	$-0.521$(14)
0.129517875	2.3144	0.96948(3)	1.663(10)	$-0.190$(18)
0.130992500	2.0298	0.91842(3)	2.696(17)	0.488(22)
0.132466875	1.7228	0.86851(3)	4.676(30)	1.963(34)
0.133941250	1.3830	0.81977(4)	9.037(65)	5.558(65)

In order to validate our computational setup, we turn to a test that is much closer to the procedure we used for the charm-quark contribution in lattice QCD. We have computed the lepton-loop contribution to $a_\mu ^{\text {HLbL}}$ using lattice fermion propagators at a mass-scale relevant for this project, choosing specifically $m_\ell /m_\mu = 20/3$. We proceed by repeating the calculation on increasingly fine lattices and perform a continuum extrapolation using a quadratic polynomial in $am_\mu$. Here, two discretisations of the vector current at the external vertex *z* were used, and the resulting contributions to $a_\mu ^\mathrm{LbL}$ were extrapolated simultaneously to the continuum, enforcing a common continuum value. The deviation of the continuum-extrapolated result from the known exact result of $16.395..\times 10^{-11}$ depends somewhat on the choice of the extrapolation range, but is in all cases within 2.5%. This successfully passed test gives us confidence that the setup used for the lattice-QCD calculation presented in the main text is robust for fermion masses up to at least 700 MeV.

## Appendix B: Tables of data for the connected contribution

This appendix contains the Tables 3 through 8 providing the results for the connected charm contribution to $a_\mu ^\mathrm{HLbL}$, using the ‘local-local’ [ll] and ‘local-conserved’ [lc] discretisations.

**Table 6:** Same as Table 3 but for ensemble N202

$\kappa _c$	$M_{\eta _c}$ [GeV]	$Z_V^{(c)}$	$a_\mu ^{\text {(Conn.[lc])}}$	$a_\mu ^{\text {(Conn.[ll])}}$
0.129934250	2.5763	9.68482(3)	1.483(7)	$-0.021$(10)
0.131111875	2.3077	0.92860(3)	2.199(10)	0.444(12)
0.132289500	2.0227	0.88955(6)	3.409(16)	1.322(16)
0.133467125	1.7154	0.85096(2)	5.650(28)	3.094(26)
0.134644750	1.3760	0.81322(2)	10.381(55)	7.115(55)

**Table 7:** Same as Table 3 but for ensemble N300

$\kappa _c$	$M_{\eta _c}$ [GeV]	$Z_V^{(c)}$	$a_\mu ^{\text {(Conn.[lc])}}$	$a_\mu ^{\text {(Conn.[ll])}}$
0.131824250	2.5779	0.92043(8)	2.066(12)	0.695(15)
0.132686875	2.3074	0.89253(4)	2.943(17)	1.361(19)
0.133549500	2.0209	0.86506(3)	4.390(27)	2.529(28)
0.134412125	1.7137	0.83794(3)	6.980(47)	4.733(44)
0.135274750	1.3763	0.81118(3)	12.236(95)	9.425(81)

**Table 8:** Same as Table 3 but for ensemble J500

$\kappa _c$	$M_{\eta _c}$ [GeV]	$Z_V^{(c)}$	$a_\mu ^{\text {(Conn.[lc])}}$	$a_\mu ^{\text {(Conn.[ll])}}$
0.132950861040	2.5849	0.89274(1)	2.245(19)	0.950(20)
0.133601050870	2.3090	0.87241(1)	3.239(27)	1.781(28)
0.134251240700	2.0194	0.85228(1)	4.860(45)	3.183(43)
0.134901430525	1.7102	0.83240(4)	7.686(77)	5.707(74)
0.135551620350	1.3718	0.81266(1)	13.255(196)	10.890(197)

## Appendix C: Fit results for the connected contribution

In this appendix we collect details of the fit results to the local-conserved connected data obtained with the fit ansätze of Eq. (8). The values obtained for $a_\mu ^\mathrm{HLbL,c}{}^\mathrm{{,conn.}}$, as well as the corresponding $\chi ^2/\text {dof}$ and *p* values are given in Table 9.

**Table 9:** Fit results of the local-conserved connected charm contribution to $a_\mu$

Fit	Dataset	$a_\mu ^\mathrm{HLbL,c}{}^\mathrm{{,conn.}}\times 10^{11}$	$\chi ^2/\text {dof}$	*p*-value
Fit1	D1	2.71(3)	1.08	0.37
Fit1	D2	2.71(3)	1.18	0.30
Fit1	D3	2.64(4)	1.07	0.38
Fit1	D4	2.76(3)	1.27	0.23
Fit2	D1	2.77(3)	1.11	0.34
Fit2	D2	2.81(3)	1.37	0.19
Fit2	D3	2.72(3)	1.14	0.33
Fit2	D4	2.85(3)	1.03	0.42
Fit3	D1	2.77(5)	1.59	0.07
Fit3	D2	2.72(5)	1.98	0.03
Fit3	D3	2.84(4)	1.74	0.07
Fit3	D4	2.66(5)	1.18	0.30
Fit4	D1	3.43(3)	1.51	0.09
Fit4	D2	3.47(3)	1.48	0.14
Fit4	D3	3.43(3)	1.90	0.05
Fit4	D4	3.45(3)	1.97	0.03
Fit5	D1	3.43(3)	1.48	0.10
Fit5	D2	3.47(3)	1.48	0.14
Fit5	D3	3.42(3)	1.92	0.04
Fit5	D4	3.45(3)	1.94	0.03

## Footnotes

1 In that paper, the $Z_V^c$ values quoted in its Appendix A are erroneously described as stemming from the charm number of the $D_s$ meson.

2 See however the text below Eq. (4) for references to the precise formulae used in the present calculation.

3 We have also investigated the effect of choosing $\Lambda =0$ and found it to make very little difference.

4 The analysis of this reference can be applied here due to the *u*, *d*, *s* quarks being degenerate and the charm quark being quenched.

5 Note that the $D^0$ loop contributes to $a_\mu ^{2+2:lc}$, even though at the scalar-QED level it does not contribute to $a_\mu ^\mathrm{HLbL}$, due to it cancelling between different topologies.

6 This picture holds when the vertices of the light-quark loop are at a distance much greater than $(2m_c)^{-1}$ from the charm loop.

7 Within the scalar QED framework, the two topologies by themselves contain equal and opposite contributions from the *D* meson loops, since they cancel in $\langle {\bar{c}} \gamma _\mu c\; {\bar{u}} \gamma _\nu u\;{\bar{u}} \gamma _\rho u\; \sum _{f=u,d,s,c} {\bar{q}}_f \gamma _\lambda q_f \rangle$, given that the charge of *D* mesons is zero under the total quark number current $\sum _{f=u,d,s,c} {\bar{q}}_f \gamma _\lambda q_f$.

8 A definition of the local and the symmetrised-conserved current can be found for instance in Ref. [42].
